# Parents’ Perceptions of Skin Cancer Threat and Children’s Physical Activity

**DOI:** 10.5888/pcd9.110345

**Published:** 2012-08-30

**Authors:** Alexander D. Tran, Jenny Aalborg, Nancy L. Asdigian, Joseph G. Morelli, Stefan T. Mokrohisky, Robert P. Dellavalle, Marianne Berwick, Neil F. Box, Lori A. Crane

**Affiliations:** Author Affiliations: Alexander D. Tran, Jenny Aalborg, Nancy L. Asdigian, Lori A. Crane, Colorado School of Public Health, University of Colorado Anschutz Medical Campus, Aurora, Colorado; Joseph G. Morelli, School of Medicine, University of Colorado Anschutz Medical Campus and Children’s Hospital Colorado, Aurora, Colorado; Neil F. Box, School of Medicine, University of Colorado Anschutz Medical Campus, Aurora, Colorado; Stefan T. Mokrohisky, Kaiser Foundation Health Plan of Colorado, Denver, Colorado; Robert P. Dellavalle, Colorado School of Public Health and School of Medicine, University of Colorado Anschutz Medical Campus, Aurora, Colorado, and Denver Department of Veterans Affairs Medical Center, Denver, Colorado; Marianne Berwick, Department of Internal Medicine and the UNM Cancer Center, University of New Mexico, Albuquerque, New Mexico.

## Abstract

**Introduction:**

Sun exposure is a major risk factor for skin cancer, but without physical activity, children are at risk of childhood obesity. The objective of this study was to explore relationships between parental perceptions of skin cancer threat, sun protection behaviors, physical activity, and body mass index (BMI) in children.

**Methods:**

This is a cross-sectional analysis nested within the Colorado Kids Sun Care Program sun safety intervention trial. In summer 2007, parent telephone interviews provided data on demographics, perceptions of skin cancer threat, sun protection behaviors, and physical activity. Physical examinations provided data on phenotype, freckling, and BMI. Data from 999 Colorado children born in 1998 were included in analysis. We used analysis of variance, Spearman’s rho (ρ) correlation, and multivariable linear regression analysis to evaluate relationships with total amount of outdoor physical activity.

**Results:**

After controlling for sex, race/ethnicity, skin color, and sun protection, regression analysis showed that each unit increase in perceived severity of nonmelanoma skin cancer was associated with a 30% increase in hours of outdoor physical activity (*P* = .005). Hours of outdoor physical activity were not related to perceived severity of melanoma or perceived susceptibility to skin cancer. BMI-for-age was not significantly correlated with perceptions of skin cancer threat, use of sun protection, or level of physical activity.

**Conclusion:**

The promotion of sun safety is not likely to inhibit physical activity. Skin cancer prevention programs should continue to promote midday sun avoidance and sun protection during outdoor activities.

## Introduction

Skin cancer is the most common form of cancer in the United States (1). Cutaneous nonmelanoma skin cancer (NMSC), including basal and squamous cell carcinoma, is the most common form of skin cancer; about 2.2 million cases are diagnosed each year (1). Cutaneous melanoma (CM) accounts for less than 5% of skin cancer cases but causes most skin cancer deaths (2). In Colorado, the rate of CM is 20% higher than the overall U.S. rate (3).

Sun exposure plays a key role in risk for skin cancer, and the extent of exposure early in life appears to influence risk of developing skin cancer later in life (4,5). The primary prevention strategy for all types of skin cancer is to reduce exposure to ultraviolet light, particularly at midday, and to use sun protection, including wearing long-sleeved shirts, long pants, and hats and applying sunscreen.

Lack of physical activity has become an important public health issue because obesity prevalence among children has nearly tripled in the United States since 1980 (6). Key strategies addressing obesity focus on encouraging children to eat a healthy diet and be more physically active. The latter strategy may conflict with skin cancer prevention efforts focused on reducing the time children spend outdoors at midday. To date, only 1 study has addressed whether promoting sun safety affects levels of physical activity or body mass index (BMI) in children (7). The study found no relationship between a sun protection intervention and either time spent outside or BMI.

According to the Health Belief Model, perceptions of susceptibility to and severity of a health problem (which together comprise the construct of “perceived threat”) are factors in predicting health behaviors (8). We hypothesized that parents with higher levels of perceived skin cancer susceptibility and severity would restrict their child’s outdoor physical activity to reduce sun exposure and the threat of skin cancer. The objective of this study was to investigate the relationships between parental perceptions of skin cancer threat, sun protection behaviors, physical activity, and BMI in children.

## Methods

### Study design

This nested cross-sectional analysis used data collected as part of the Colorado Kids Sun Care Program, a randomized controlled trial assessing sun protection practices of parents to reduce skin cancer risk for their children (9). The study follows a cohort of 1,145 children born from January through September 1998 and recruited from a large managed care organization, private pediatrician offices, and various community locations in the Denver-Boulder-Colorado Springs area of Colorado. Families were randomly selected to receive standard care or a combination of parent newsletters and child newsletters in the spring of 2005, 2006, and 2007 to encourage sun protection behaviors. Some intervention mailings also included sun protection hats, swim shirts, sunscreen, backpacks, and information on child activities related to sun safety. In summer 2007, parent telephone interviews and child physical examinations provided data. Verbal consent was accepted for telephone interviews. Parents provided written informed consent and children provided written assent for participation in physical examinations. This study was reviewed and approved by the Colorado Multiple Institutional Review Board and the Kaiser Permanente of Colorado Institutional Review Board.

The original cohort included 1,145 children. In 2007, 1,001 parents of these children (87%) completed the telephone interview. We excluded 2 children with missing physical activity data, leaving 999 for analysis. Of these, 831 completed a physical examination in 2007; thus, analyses of variables collected during examinations are limited to these participants. All children were aged 8 to 9 years in 2007.

### Measurement

#### Parent interviews

Trained staff used a computer-assisted telephone interviewing system to interview parents and legal guardians by telephone during the summer months of 2007 (June–September). Up to 20 calls were made to reach each family. Participants received $25 for completing the telephone interview.

Parents reported the child’s race and ethnicity. For analysis, categories were reduced to non-Hispanic white, Hispanic white, and other. Parent annual income responses were collapsed to “less than $75,000,” “$75,000-$99,999,” and “$100,000 or more” for analysis. Parent education level was categorized as “some college or less,” “college graduate,” and “beyond college.”

Skin cancer perception questions developed for this study were based on the Health Belief Model and the Precaution Adoption Process Model (8,10). Perceived CM severity was assessed by asking parents 7 questions. The first question was, “How serious do you think melanoma is?” Responses were recorded on a numeric scale ranging from 0 (not at all serious) to 10 (extremely serious). The next 2 questions asked parents whether it was true or false that treatment of melanoma skin cancer usually involves 1) chemotherapy and 2) removing large areas of tissue around the affected skin. Parents were also asked, “How easy or hard is it for doctors to treat a typical case of melanoma?” Responses were recorded on a 4-point scale ranging from 1 (very easy to treat) to 4 (very difficult to treat). Next, parents were asked how common it is for melanoma skin cancer to be cured if detected either early or late; responses were recorded on a 5-point scale with higher values indicating lower likelihood of cure (ie, more advanced disease). Finally, parents were asked, “How common do you think it is to die from it?” with answers obtained on a 5-point scale ranging from 1 (almost none die) to 5 (nearly everyone dies).

Perceived NMSC severity was assessed by asking parents 6 questions following the same format as for CM. The likelihood of recovering from NMSC was asked as 1 general question: “How common is it for nonmelanoma skin cancer to be cured?” instead of 2 separate questions as for CM.

For perceived CM and NMSC susceptibility, parents were asked the following question: “Thinking about your child, what you do now, and where you live, how likely do you think [CHILD’S NAME] is to get [melanoma/nonmelanoma skin cancer] over [his/her] whole life?” Responses were recorded on a 7-point scale ranging from 1 (no chance at all) to 7 (certain to happen).

Parents were asked how often their child engages in 4 sun-protective habits while outdoors on sunny days during the summer between 11 am and 3 pm: wearing clothes that cover most of the arms and legs, staying in the shade, wearing a hat, and wearing sunscreen or sunblock (11,12). Responses were recorded with a 5-point scale ranging from 1 (never) to 5 (all the time) (11,12). Sun sensitivity was assessed using a 4-category scale of the child’s expected reaction to 1 hour of strong sunshine exposure at the beginning of the summer (degree of sunburn the following day relative to the degree of tan 1 week later: painful/none, painful/light, slight/little, none/good).

Parental reports of the hours per week their child spends being physically active (in general and outdoor-specific) served as the primary outcome measures in this study. Questions were based on measures used in the Behavioral Risk Factor Surveillance System (13). Total amount of physical activity per week was obtained by asking, “In a typical week, how many hours does [CHILD] spend playing sports or doing some other physical activity like dance, roller-skating, or riding a bicycle?” (14). Total amount of outdoor physical activity was assessed by asking the number of hours per week the child spends in 5 specific outdoor activities: swim team, recreational swimming, hiking, cycling, and soccer (common outdoor activities among Colorado children). Total outdoor physical activity was calculated by summing across these 5 outdoor activities.

#### Physical examinations

Physical examinations were conducted from June through September 2007. Height and weight were measured using standard clinical procedures. Hair color was determined through the use of wigmaker samples and categorized for analysis as blonde, light brown, red, dark brown, or black (9). To increase reliability in measurement, hair color was assigned as the color recorded at most skin examinations during a 4-year period (2004–2007) or at the first skin examination if there was no majority. The same approach was used to determine a fixed eye color for each child. Using a color chart, we categorized eye color as blue, green, or brown (9). Facial freckling was assessed using a described previously 10-level chart (15). Facial freckling was collapsed to “none” vs “any.” Base skin pigmentation was measured using a colorimeter (Minolta Chroma Meter CR-400; Konica Minolta Sensing, Inc., Ramsey, New Jersey), which quantifies color using the 3-dimensional L-a-b system (16,17). The L-scale, which measures color from white to black, was used (smaller values indicate darker color) (18,19). Base skin color was dichotomized as “very fair” (L score ≥60) vs “other” (L score of <60, including darker white, brown, and black) to identify those at highest risk for skin cancer (17).

### Data analysis

Child’s BMI was calculated by using the formula of BMI equals [(weight in pounds) × 703] / [(height in inches)^2^] (20). BMI percentiles were determined by using the year 2000 Centers for Disease Control and Prevention BMI-for-age growth charts for children aged 2 to 20 years (21). Using these percentiles, we classified children as underweight (<5th percentile), healthy weight (5th-84th percentile), overweight (85th-94th percentile), or obese (≥95th percentile).

A sun protection index variable (range, 1–5) was generated by taking the mean of responses to questions about frequency of use of clothing, hats, and sunscreen. Shade was retained as a separate variable.

Total outdoor physical activity was calculated by summing the 5 individual outdoor activities described earlier. For correlation and regression analyses, the physical activity variables were natural log transformed because of positive skew.

All questionnaire items related to perceptions of skin cancer threat (described above) were recoded into 5-point scales so that all items would be equally weighted. Items were entered into a principal components analysis with varimax rotation. The exploratory factor analysis provided support for 3 distinct factors: perceived CM severity (7 items related to CM severity), perceived NMSC severity (6 items related to NMSC severity), and perceived skin cancer susceptibility (2 items related to CM and NMSC susceptibility). Three additive scales were constructed from these items. Cronbach’s alphas for the scales were 0.71, 0.56, and 0.79, respectively. The distribution of perceived CM severity was negatively skewed; therefore, values were squared. The distribution of perceived NMSC severity was positively skewed; therefore, a natural log transformation was used.

### Statistical methods

A *P* value of ≤.05 was routinely used to assign statistical significance. All statistical analyses were carried out by using SPSS version 20 (IBM, Chicago, Illinois) software. Mean total amount of time in all physical activity (hours/week) and outdoor physical activity (hours/week) were compared within phenotype and demographic subgroups variables by using 1-way analysis of variance. Komolgorov-Smirnov test was used to compare the distribution of perceived susceptibility to CM with perceived susceptibility to NMSC. Spearman’s rho (ρ) correlation was used to assess associations between perceptions of skin cancer threat, sun protection behaviors, physical activity levels, and BMI-for-age percentile. Multiple linear regression analysis was used to evaluate an identified association between perceived NMSC severity and total amount of outdoor physical activity, using a backward modeling approach. All variables associated with outdoor activity at a level of *P* < .25 in descriptive analyses were included in initial modeling. Intervention status was included to ensure that any relationships with physical activity were independent of study group assignment. The least significant predictors were eliminated sequentially until all retained variables were significant at *P* < .15, with the exception of intervention status. After each elimination step, regression coefficients were checked to confirm that confounding was not occurring. Regression coefficients were back-transformed by taking the antilog so that they represent the percentage change in hours of physical activity associated with a unit increase on each predictor variable.

## Results

We analyzed the characteristics of the participants included in this analysis and the relationships between these characteristics and hours of total physical activity and hours of total outdoor physical activity (Table 1). The sample was approximately evenly distributed between boys and girls; boys had a significantly higher level of total physical activity (*P* < .001) but not outdoor physical activity. Most (80.6%) of the children in this study were non-Hispanic white. Hispanic white children were reported to engage in more total physical activity (*P* = .04) and outdoor physical activity (*P* = .03) compared with non-Hispanic white children. The lowest levels of physical activity were reported for children in the “other” category. Slightly less than one-third of the children had “very fair” skin; children with “other” base skin color reported a significantly higher amount of both all physical activity and outdoor physical activity (*P* < .001). Parents perceived a greater likelihood that their child would get NMSC compared with CM.

**Table 1:** Characteristics of Study Participants and Hours of Physical Activity, Children (n = 999) Aged 8–9 Years, Colorado, 2007

Characteristic	Amount of Time in Physical Activity, All Types (h/wk)				Amount of Time in 5 Outdoor Physical Activities^a^ (h/wk)	
	n	%^b^	Mean (SD)	*P* Value^c^	Mean (SD)	*P* Value^c^
**Sex**						
Female	509	51.0	19.4 (11.7)	<.001	10.9 (8.0)	.21
Male	490	49.0	22.3 (13.0)		11.6 (8.4)	
**Race/ethnicity^d^**						
Non-Hispanic white	804	80.6	20.8 (12.1)	.04	11.1 (7.9)	.03
Hispanic white	137	13.7	22.6 (14.3)		12.7 (10.0)	
Other	57	5.7	17.6 (12.2)		9.5 (7.3)	
**Parent annual income^e^, $**						
<75,000	407	42.3	20.8 (13.5)	.43	11.0 (8.5)	.19
75,000–99,999	258	26.8	20.3 (11.7)		10.9 (8.0)	
≥100,000	297	30.9	21.6 (11.5)		12.0 (8.0)	
**Parent education**						
Some college or less	280	28.0	20.9 (13.4)	.26	11.0 (8.9)	.83
College graduate	415	41.6	20.2 (12.2)		11.3 (8.0)	
Beyond college	304	30.4	21.7 (11.8)		11.4 (7.9)	
**Hair color^f^**						
Blonde	83	8.7	22.3 (13.0)	.30	11.6 (7.7)	.62
Light brown	368	38.5	20.7 (12.8)		11.2 (8.3)	
Red	39	4.1	17.8 (10.1)		9.2 (7.5)	
Dark brown	413	43.3	20.9 (12.1)		11.2 (8.1)	
Black	52	5.4	18.8 (11.9)		10.6 (8.5)	
**Eye color^f^**						
Blue	300	31.4	21.2 (13.0)	.72	11.6 (8.4)	.38
Green	325	34.0	20.4 (11.2)		10.7 (7.3)	
Brown	330	34.6	20.6 (12.9)		11.1 (8.7)	
**Presence of freckling^g^**						
None	265	32.0	20.0 (13.0)	.32	10.8 (8.5)	.44
Any	564	68.0	21.0 (12.2)		11.2 (8.0)	
**Base skin color^g^**						
Other (<60)	578	69.7	21.7 (12.9)	<.001	11.9 (8.5)	<.001
Very fair (≥60)	251	30.3	18.3 (10.9)		9.1 (6.7)	
**Sun sensitivity^h^**						
Painful burn/no tan (type 1)	94	9.4	19.3 (12.3)	.33	10.6 (8.4)	.47
Painful burn/light tan (type 2)	222	22.3	20.4 (12.0)		10.9 (8.0)	
Slight burn/little tan (type 3)	454	45.5	20.1 (12.5)		11.2 (7.9)	
No burn/good tan (type 4)	227	22.8	21.9 (12.7)		11.9 (8.9)	
**BMI-for-age percentile^i^**						
Underweight	41	4.9	18.6 (11.9)	.31	8.6 (6.9)	.16
Healthy weight	645	77.8	21.1 (12.7)		11.4 (8.2)	
Overweight	89	10.7	19.3 (11.9)		10.7 (8.3)	
Obese	55	6.6	19.3 (10.6)		10.5 (8.2)	
**Intervention status**						
Control	373	37.3	20.9 (12.0)	.89	11.3 (8.1)	.73
Intervention	626	62.7	20.8 (12.7)		11.2 (8.3)	
**Parents’ perception of likelihood that their child will get melanoma in the future^j^**						
Not at all likely-not very likely	634	63.7	20.2 (11.8)	.12	10.8 (8.0)	.15
50/50 chance-very likely	361	36.3	22.0 (13.3)		12.0 (8.6)	
**Parents’ perception of likelihood that their child will get nonmelanoma skin cancer in the future^j^**						
Not at all likely-not very likely	548	55.4	20.3 (12.1)	.18	11.0 (8.2)	.23
50/50 chance-very likely	442	44.6	21.4 (12.7)		11.5 (8.2)	

Abbreviation: BMI, body mass index.

^a^ The 5 outdoor activities were swim team, recreational swimming, hiking, cycling, and soccer.

^b^ Percentage of valid responses.

^c^ Differences between groups assessed by using analysis of variance, and *P* ≤ .05 was used to assign significance.

^d^ Race/ethnicity was not reported by 1 participant; “other” category includes 25 blacks/African Americans, 28 Asian/Pacific Islanders, and 4 American Indians/Alaska Natives.

^e^ Parent income was not reported by 37 respondents.

^f^ Hair and eye color were unavailable for 44 participants.

^g^ Presence of freckling and base skin color were unavailable for 170 participants; L-scale was used to measure base skin color (18,19).

^h^ Sun sensitivity was not reported by 2 participants.

^i^ BMI-for-age percentile was unavailable for 169 participants. BMI-for-age percentile was categorized as follows: underweight, BMI <5th percentile for age; healthy weight, BMI 5th-84th percentile for age; overweight, BMI 85th-94th percentile for age; and obese, BMI ≥95th percentile for age.

^j^ Likelihood of getting melanoma was not reported by 4 participants; chance of getting nonmelanoma skin cancer was not reported by 9 participants; difference in distribution between these 2 variables was tested using Komolgorov-Smirnov test. Distributions were different at *P* < .001.

We found no significant associations between perceived CM severity and the total amount of physical activity, total outdoor physical activity, or BMI-for-age percentile (Table 2). Perceived NMSC severity was positively and significantly related to total physical activity (*P* = .007) and the total of 5 outdoor physical activities (*P* = .002) but not related to BMI-for-age percentile. Perceived skin cancer susceptibility was not related to either of the physical activity variables or BMI-for-age percentile.

**Table 2:** Spearman’s Rho (ρ) Correlations for Relationships of Physical Activity and BMI With Skin Cancer Threat Perceptions and Sun Protection Behaviors, Children (n = 999) Aged 8–9 Years, Colorado, 2007

Variables	Perceived Melanoma Severity^a^		Perceived Nonmelanoma Severity^b^		Perceived Skin Cancer Susceptibility		Use of Shade		Sun Protection Index^c^	
	ρ^d^	*P* Value^e^	ρ^d^	*P* Value^e^	ρ^d^	*P* Value^e^	ρ^d^	*P* Value^e^	ρ^d^	*P* Value^e^
Total physical activity^f^ (h/wk)	−.002	.96	.086	.007	.040	.21	−.096	.003	.048	.14
Total of 5 outdoor physical activities^g^ (h/wk)	.016	.62	.097	.002	.025	.43	−.083	.01	.026	.42
BMI-for-age percentile	.011	.75	.039	.27	.014	.68	.022	.55	−.001	.97

Abbreviation: BMI, body mass index.

^a^ Variable was squared to adjust for negatively skewed distribution.

^b^ Variable was natural log transformed to adjust for positively skewed distribution.

^c^ Sun protection index composed of parents’ reports of child’s use of clothing, hat, and sunscreen.

^d^ Spearman’s (ρ) correlation coefficient.

^e^ *P* value based on Spearman’s rho (ρ) correlation coefficient, and *P* ≤ .05 was used to assign significance.

^f^ Natural log transformed to adjust for positively skewed distribution and used as a continuous variable in analysis.

^g^ Natural log transformed to adjust for positively skewed distribution and used as a continuous variable in analysis. The 5 outdoor activities were swim team, recreational swimming, hiking, cycling, and soccer.

Use of shade for sun protection exhibited a significant negative relationship with total physical activity (*P* = .003) and outdoor physical activity (*P* = .01), but the sun protection index was not related to levels of physical activity. Neither shade nor sun protection presented a significant correlation with BMI-for-age percentile.

We used multivariable linear regression to assess relationships with outdoor physical activity (Table 3). The anti-log transformations of regression coefficients represent the multiplicative factors by which hours of outdoor physical activity change for every unit increase in the predictor variable. Each unit increase in perceived NMSC severity (natural log-transformed) was associated with a 30% increase in hours of outdoor physical activity (*P* = .005). Each unit increase in the sun protection index was associated with a 9% increase in outdoor physical activity (*P* = .03). Each unit increase in shade-seeking was associated with a 7% decrease in outdoor physical activity (*P* = .02). Finally, children with very fair skin reported 25% fewer hours of outdoor physical activity than did children with other skin colors (*P* < .001).

**Table 3:** Predictors of Outdoor Physical Activity, Children (n = 999) Aged 8–9 Years, Colorado, 2007

Characteristic^a^	Unstandardized Coefficient^b^ (β)	Standard Error	P Value^c^	Antilog^d^ (β)
**Sex**				
Male	1 [Reference]		1 [Reference]	
Female	−.098	.055	.08	.91
**Base skin color^e^**				
Other (<60)	1 [Reference]		1 [Reference]	
Very fair (≥60)	−.281	.060	<.001	.76
**Race/ethnicity^f^**				
White non-Hispanic	1 [Reference]		1 [Reference]	
White Hispanic	−.111	.082	.18	.89
Other	−.173	.117	.14	.84
**Perceived nonmelanoma severity^g^**	.260	.092	.005	1.30
**Sun protection index^h^**	.088	.041	.03	1.09
**Use of shade**	−.075	.033	.02	.93
**Intervention status**	.001	.054	.98	1.00

Abbreviation: BMI, body mass index.

^a^ Predictor variables in final regression model; BMI-for-age and parent income were also initially included but were removed due to lack of contribution to model.

^b^ Unstandardized coefficient for natural log transformed outcome variable: hours per week in 5 outdoor physical activities. The 5 outdoor activities were swim team, recreational swimming, hiking, cycling, and soccer.

^c^ *P* value based on multiple linear regression, and *P* ≤ .05 was used to assign significance.

^d^ Multiplicative factor by which hours of outdoor physical activity change for every unit increase in predictor.

^e^ L-scale was used to measure base skin color (18,19).

^f^ “Other” category includes black/African American, Asian/Pacific Islander, and American Indian/Alaska Native.

^g^ Natural log transformed to adjust for positively skewed distribution and used as a continuous variable in regression analysis.

^h^ Sun protection index composed of parents’ reports of child’s use of clothing, hat, and sunscreen.

## Discussion

Contrary to our hypothesis, this study found a positive relationship between perceived NMSC severity and levels of total physical activity and outdoor physical activity among Colorado children aged 8 or 9. Furthermore, higher levels of outdoor physical activity were associated with more frequent use of sun protection. The findings suggest an alternative explanation: that parents whose children engage in higher levels of outdoor physical activity have a heightened awareness of skin cancer severity and therefore use more sun protection. This is logical because children who spend more time outside are at greater risk for skin cancer later in life (5). The finding that perceived severity of NMSC, but not CM, was related to outdoor physical activity is somewhat surprising since CM is a much more severe disease, generally requiring more extensive treatment and having a higher probability of death. The much greater incidence of NMSC (1,22) may make it more prominent in parents’ minds when formulating perceptions of skin cancer threat and making decisions about sun protection. Because of its greater incidence, parents are more likely to know someone who has had NMSC than CM. Our data show that parents perceived higher chances that their child will get NMSC than CM (Table 1), which supports this explanation. The thought processes of parents regarding skin cancer threat, outdoor activity, and sun protection might be the subject of future qualitative studies.

We found that BMI-for-age was not related to perceptions of skin cancer threat, use of sun protection, total physical activity, or outdoor physical activity. BMI is related to both food intake and physical activity, and our study did not assess food intake. Only 7% of our sample were classified as obese, whereas almost 20% of children aged 6 through 11 nationwide were classified as obese in 2007 (23). Our study cohort is relatively affluent, which may explain why the distribution of BMI is different than that of the general population; this may limit the generalizability of the findings. Although these are important limitations, the lack of relationship between sun safety variables and BMI in our study is in agreement with the only other study to examine this issue (7). Our study is also limited because the cross-sectional design does not allow us to determine cause and effect. Furthermore, our measure of outdoor physical activity most likely underestimates total outdoor physical activity because it represents only hours spent in 5 specific activities and excludes other activities, including unstructured play. Our study relied on parents to report behaviors, which is subject to response bias (24).

To our knowledge, this analysis is the first to examine the relationship between perceptions of skin cancer threat and physical activity. The study was conducted in a location that gets high sun exposure because of climate and altitude and has melanoma rates that are approximately 20% higher than those for the United States as a whole (3). The size of our cohort is an additional strength.

Two studies conducted in different settings now suggest that the promotion of sun safety is not likely to inhibit outdoor physical activity. Although future studies should clarify and confirm these findings, this is encouraging for both sun safety and obesity prevention efforts. Skin cancer prevention efforts should be continued by focusing on using good sun protection practices while engaging in outdoor physical activity.
